# Gastroesophageal Reflux Disease Relief in Patients Treated with Rabeprazole 20* *mg versus Omeprazole 20* *mg: A Meta-Analysis

**DOI:** 10.1155/2013/327571

**Published:** 2013-09-09

**Authors:** X. M. Xia, H. Wang

**Affiliations:** The First Affiliated Hospital of Anhui Medical University, 218 Jixi Road, Hefei, Anhui 230022, China

## Abstract

*Background*. Randomized controlled trials (RCTs) have been conducted comparing the efficacy of rabeprazole 20 mg or omeprazole 20 mg once daily for patients with erosive gastroesophageal reflux disease (GERD). Until now, no study has synthesized all available data examining this issue. *Method*. Medline, Embase, and the Cochrane central register of controlled trials were searched (through December 2012). Eligible RCTs recruited adults with erosive GERD and reported endoscopic and symptomatic relief rates at the last point of follow-up. The effect of rabeprazole versus omeprazole was reported as relative risk (RR) of relief with a 95% confidence interval (CI). *Results*. The search identified 605 citations, and six RCTs containing 1,895 patients were eligible. Endoscopic relief rates were not significantly different between rabeprazole 20 mg and omeprazole 20 mg in treatment trials of up to 8 weeks. Heartburn relief rates were significantly different between the two groups for 8-week treatment trials. Adverse events were not significantly different between the two groups for 8-week treatment trials. *Conclusion*. These data suggest that rabeprazole demonstrates a clinical advantage over omeprazole in symptomatic relief but no significant difference in endoscopic relief of erosive GERD for up to 8 weeks of treatment. Rabeprazole and omeprazole were both tolerated by GERD patients.

## 1. Introduction

Gastroesophageal reflux disease (GERD) is a recurrent chronic disorder characterized by increased reflux of gastric contents into the lower esophagus that affects approximately 20%–30% of the population worldwide, particularly in western countries [1–3]. Severe reflux esophagitis may develop complications such as esophageal stricture or Barrett's esophagus [4]. Previous clinical studies have shown that proton pump inhibitors (PPIs) are safer and more effective than H_2_ receptor antagonists at healing esophageal lesions, relieving heartburn symptoms, and preventing symptomatic and endoscopic relapse [5, 6]. Omeprazole and rabeprazole are both potent inhibitors of H^+^K^+^-ATPase, which is responsible for the terminal step in gastric acid secretion [7]. Rabeprazole is a second-generation proton pump inhibitor that has 2- to 10-fold greater antisecretory activity *in vitro* than omeprazole, the prototypical PPI [8–11]. A rapid pharmacodynamic response may translate to faster onset of symptom relief [9]. However, meta-analysis by Caro et al. showed that rabeprazole was of similar efficacy to omeprazole in terms of heartburn control, healing rates, and relapse rates [5]. Other head-to-head clinical trials have been carried out demonstrating statistically significant and clinically relevant differences in healing efficacy and symptomatic relief of erosive GERD between rabeprazole 20 mg and omeprazole 20 mg daily dosing, but available data examining this issue also reveal inconsistent results [7, 12–16].

Meta-analysis is an accepted methodology that increases the ability to detect small yet statistically significant and perhaps clinically relevant differences. Previous meta-analyses examining the efficacy of rabeprazole versus omeprazole in treating erosive GERD included only two relevant randomized controlled trials (RCTs) [5]. However, relying on underpowered comparative studies might lead to type II error, in which a true difference between agents cannot be detected because of the inadequate sample size [17]. We therefore conducted an updated systematic review and meta-analysis of published RCTs of rabeprazole 20 mg versus omeprazole 20 mg dosing to evaluate healing rates and symptom relief in erosive GERD. 

## 2. Materials and Methods

### 2.1. Search Strategy

We investigated published work, without language restriction, using Medline (January 1966 to December 2012), Embase (January 1980 to December 2012), Web of Science (1994 to December 2012), and the Cochrane Central Register of Controlled Trials (issue 12, 2012). The following keywords were used: esophagitis, reflux disease, GERD, omeprazole, and rabeprazole. 

### 2.2. Eligibility Criteria

We included RCTs involving patients and comparing rabeprazole 20 mg once daily with omeprazole 20 mg once daily for maintenance therapy lasting up to 8 weeks. Studies assessed healing of erosive GERD endoscopically using Hetzel-Dent (HD), Savary-Miller (SM), and Los Angeles (LA) classifications. Studies of 1-week treatment of GERD with rabeprazole 20 mg versus omeprazole 20 mg once daily, using symptomatic relief of erosive GERD as a criterion for efficacy, were also included in the study. Patients included had to be older than 18 years. Studies without raw data and duplicate publications were not eligible.

### 2.3. Data Extraction

We extracted from each article author information, year of publication, type of study, country of origin, study population, sex, sample size, criteria for inclusion and exclusion, method of randomization, adequacy of concealment of allocation, details of blinding and outcome assessments, type and dose of medication, length of treatment, grading system for esophagitis (SM, HD, LA, or their modifications), number of intention-to-treat (ITT) patients in each study arm, healing data in each study arm, justification for dropping out, and criteria defining healing or relief. The main efficacy outcomes pooled in this analysis include the symptomatic relief rate and the endoscopic relief rate.

### 2.4. Statistical Analysis

Healing of esophagitis was confirmed using endoscopy. The primary analysis of this study was to compare the rate of endoscopic relief between the groups treated with rabeprazole 20 mg or omeprazole 20 mg. The secondary analysis was to compare the rate of symptomatic relief (mainly heartburn relapse) between the two groups. The third analysis was to compare the rate of adverse events between the two groups. Relative risk (RR) was used as a measurement of the relationship between PPI therapy and the risk of GERD relief. Differences between groups were expressed as RR with 95% confidence interval (CI). Individual RR and 95% CI were extracted or calculated initially. The fixed-effect model and the random-effect model were used, with the significance level set at *P* < 0.05. Statistical heterogeneity between trials was evaluated using the *I* ^2^ test. In the presence of statistical heterogeneity, the random-effect model was used for the analysis. In the absence of statistically heterogeneity, the RR using the fixed-effect model was calculated. The presence of publication bias was assessed by examining a funnel plot and by calculating the Egger coefficient. All analyses were performed on an ITT basis, which was defined as the inclusion of all patients whether or not they received treatment. The meta-analyses were conducted using Stata 11.0 (Stata Corporation, Lakeway, TX, USA).

## 3. Results

**Figure 1:**
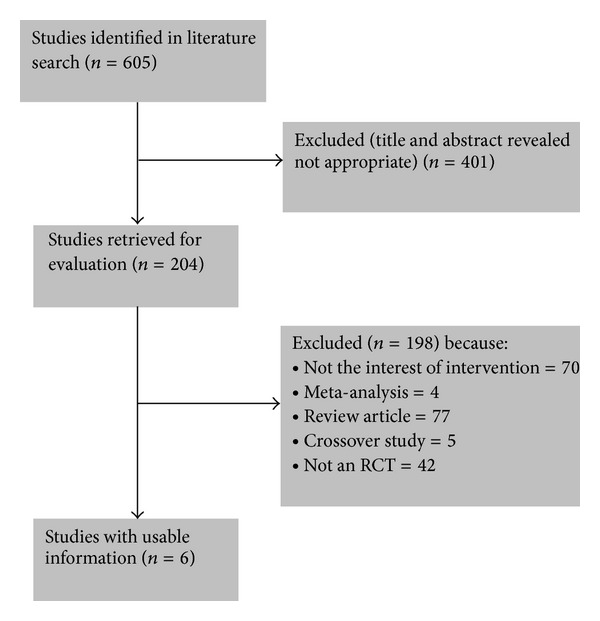
Flow diagram of assessment of studies identified in the systematic review. RCT: randomized controlled trial.

The search strategy summarized in Figure 1 identified 605 citations, 401 of which were excluded after examining the title and abstract. A total of 204 articles reporting on the efficacy of rabeprazole or omeprazole in GERD were retrieved and evaluated in more detail. Of these, 198 were excluded for various reasons, leaving six RCTs that were eligible for inclusion (Figure 1) [7, 12–16]. All of the trials recruited patients with a previous diagnosis of erosive GERD that had been healed within 90 days before study entry [7, 12–16]. All six RCTs recruited individuals with GERD for treatment with rabeprazole 20 mg or omeprazole 20 mg [7, 12–16]. Duration of treatment and follow-up was 1 week in one trial, and 8 weeks in the other five trials. The characteristics of the original studies are presented in Table 1. All six studies were conducted in Europe and Japan. 

### 3.1. Endoscopic Relief of GERD

**Figure 2:**
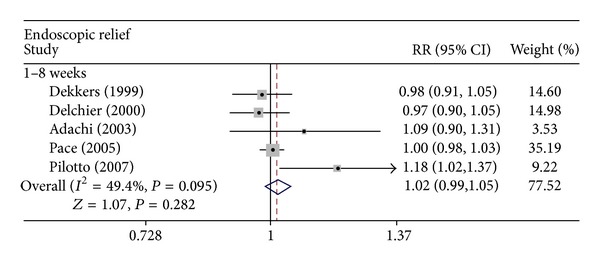
Effect of rabeprazole 20 mg once daily versus omeprazole 20 mg once daily on endoscopic relief of GERD. RR, relative risk; CI, confidence interval.

The six trials contained a total of 1,895 patients who received up to 8 weeks of maintenance treatment [7, 12–16]. The primary analysis of the present study was the comparison of the rates of endoscopic relief between groups treated with rabeprazole 20 mg or omeprazole 20 mg. Endoscopic relief rates were not significantly different between the two groups for up to 8 weeks of treatment (RR = 1.018; 95% CI: 0.986–1.050; *P* = 0.282), with no heterogeneity between studies (*I* ^2^ = 49.4%; *P* = 0.095) (Figure 2). The present study revealed no publication bias (Egger test, *P* = 0.133) and no significant difference in endoscopic relief of erosive GERD between the two groups.

### 3.2. Relief of GERD-Related Heartburn

**Figure 3:**
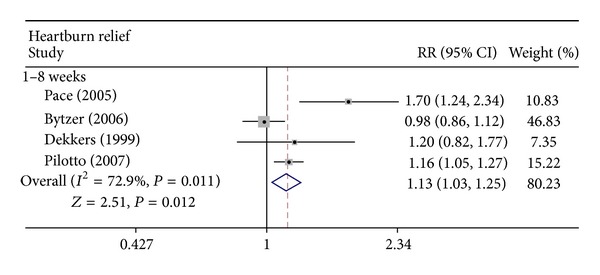
Effect of rabeprazole 20 mg once daily versus omeprazole 20 mg once daily on GERD-related heartburn relief. RR: relative risk; CI: confidence interval.

The secondary analysis of this study was comparison of the rates of symptomatic relief (mainly heartburn relief) between the two groups. A statistically significant difference was detected in heartburn relief between rabeprazole 20 mg and omeprazole 20 mg once daily for up to 8 weeks of treatment (RR = 1.133; 95% CI: 1.028–1.249; *P* = 0.012), as well as evidence of statistical heterogeneity (*I* ^2^ = 72.9%, *P* = 0.011) (Figure 3). Publication bias was not observed (Egger test, *P* = 0.060). Analyses of the above trials favored rabeprazole 20 mg over omeprazole 20 mg for relief of heartburn in erosive GERD.

### 3.3. Adverse Events

**Figure 4:**
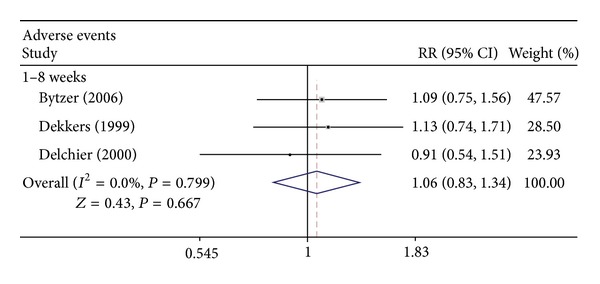
Adverse events of rabeprazole 20 mg once daily versus omeprazole 20 mg once daily in the treatment of GERD. RR: relative risk; CI: confidence interval.

The third analysis of this study was a comparison of the rates of adverse events between the two groups. Three RCTs containing raw data of adverse events were analyzed. No statistically significant difference in adverse events was detected between rabeprazole 20 mg once daily and omeprazole 20 mg once daily for up to 8 weeks of treatment (RR = 1.055; 95% CI: 0.827–1.345; *P* = 0.667), with no evidence of statistical heterogeneity (*I* ^2^ = 0%, *P* = 0.799) (Figure 4). Publication bias was observed in the study (Egger test, *P* = 0.027). Analyses of the included trials showed no significant difference in adverse events between the two groups for treatment of erosive GERD.

## 4. Discussion

This systematic review and meta-analysis demonstrates that rabeprazole 20 mg is more effective than omeprazole 20 mg for the relief of heartburn symptoms in trials evaluating up to 8 weeks of GERD treatment. Meta-analysis shows no significant difference in endoscopic relief of erosive GERD between rabeprazole 20 mg and omeprazole 20 mg for up to 8 weeks of treatment. The eligible trials we identified recruited > 1,800 patients, meaning that any difference in effect between rabeprazole 20 mg and omeprazole 20 mg is likely to be small. Adherence to medication was not different between treatment arms in the six trials, all of which reported data concerning compliance with therapy [7, 12–16]. 

PPIs such as rabeprazole and omeprazole are in widespread use to heal esophageal lesions and relieve heartburn symptoms. Rabeprazole is a substituted benzimidazole derivative and is structurally related to omeprazole. However, in preclinical experiments, rabeprazole was shown to be more rapid and potent than omeprazole in inhibiting H^+^K^+^-ATPase [11]. This rapid pharmacodynamic response may translate into faster onset of symptom relief [9]. RCTs from Adachi et al., Pace et al., and Pilotto et al. revealed that, for trials of up to 8 weeks, rabeprazole was more effective than omeprazole for the rapid relief of heartburn symptoms in patients with reflux esophagitis [13, 14, 16]. However, inconsistent results from RCTs demonstrate that rabeprazole 20 mg was as effective as omeprazole 20 mg in relieving heartburn symptoms of GERD in trials of 1 week [12, 15] and 8 weeks [7] of treatment. In the present study, meta-analysis revealed that in trials of up to 8 weeks of treatment, rabeprazole 20 mg is more effective than omeprazole 20 mg in relieving the GERD symptom of heartburn. Results from the present study favor rabeprazole 20 mg over omeprazole 20 mg in the relief of GERD-related heartburn.

To define further the efficacy of rabeprazole 20 mg and omeprazole 20 mg at relieving symptoms of GERD, we also performed an analysis of endoscopic relief after therapy. After 8 weeks of treatment, four RCTs demonstrated that rabeprazole 20 mg was equivalent to omeprazole 20 mg in endoscopic relief of erosive GERD [7, 12–14]. However, one RCT, conducted by Pilotto et al., demonstrated that rabeprazole was significantly more effective than omeprazole at healing erosive GERD [16]. In this study, meta-analysis of the included RCTs showed that rabeprazole 20 mg is as effective as omeprazole 20 mg with regard to endoscopic relief of erosive GERD for up to 8 weeks of treatment. 

Safety is always a concern in PPI administration. Short-term treatment of GERD with omeprazole or rabeprazole is well tolerated by patients, and treatment-related adverse events are relatively few [18–20]. Similar safety profiles were seen in the RCTs included in this study. Bytzer et al. demonstrated that PPI treatment for 1 week was well tolerated, and the incidence of adverse events following treatment with rabeprazole (14.5%) was similar to that following omeprazole treatment (13.4%) [15]. RCTs conducted by Dekkers et al. and Delchier et al. showed no significant difference between rabeprazole and omeprazole groups in the incidence of adverse events in 8-week trials [7, 12]. In the present study, our meta-analysis revealed that treatment-related adverse events were not significantly different between rabeprazole 20 mg and omeprazole 20 mg for treatment trials up to 8 weeks. These results revealed that rabeprazole and omeprazole were both tolerated by GERD patients. 

The present analysis has several strengths. First, we used a standardized and systematic search strategy, including both Medline and Embase, to identify relevant studies. Second, analyses were performed in parallel by two abstractors blinded to each other's status. Third, the studies in our meta-analysis included a considerable number of subjects (*n* = 1, 895) and did not show evidence of publication bias in primary analyses of efficacy. 

There are, however, also limitations to this study. First, the included studies were conducted only in Europe and Japan; no studies conducted in other countries were found. Second, not all studies reported rates of adverse events, precluding any pooling of data for these outcomes. Third, there was evidence of publication bias in primary analyses of adverse events. The absence of negative or inconclusive data could produce a misleading bias in the overall published literature [21]. The Egger test not only reflects publication bias, but also describes small study effects. Because of the limitations of our meta-analysis, further studies are required to identify the clinical importance of the present findings. Well-designed, large-scale RCTs would be preferred [22]. RCTs should also be conducted worldwide, with subjects of different races. 

In summary, these data suggest a clinical advantage of rabeprazole over omeprazole in symptomatic relief, but no significant difference in endoscopic relief, of erosive GERD for up to 8 weeks of treatment. Rabeprazole and omeprazole were both tolerated by GERD patients. 

## Figures and Tables

**Table 1:** Characteristics of the included studies.

Source	Study design	Region	Identification of relief	Number of patients		Mean ages (years)	Daily dosage		Duration
				Male	Female		RAB 20 mg	OME 20 mg	
Dekkers et al. (1999) [7]	DB, RCT	Europe, 27 centers	Endoscopy, heartburn symptom	126	76	53	100	102	8 weeks
Delchier et al. (2000) [12]	DB, RCT	Europe, 50 centers	Endoscopy, heartburn symptom	87	120	54	104	103	8 weeks
Adachi et al. (2003) [13]	DB, RCT	Japan, 6 centers	Endoscopy, heartburn symptom	30	30	66	30	30	8 weeks
Pace et al. (2005) [14]	DB, RCT	Italy, 71 centers	Endoscopy, heartburn symptom	374	175	47	277	272	8 weeks
Bytzer et al. (2006) [15]	DB, RCT	Europe, 49 centers	Heartburn symptom	374	343	51	358	359	1 week
Pilotto et al. (2007) [16]	Unblind, RCT	Italy, 1 center	Endoscopy, heartburn symptom	81	79	77	80	80	8 weeks

DB: double-blind; RCT: randomized clinical trial; RAB: rabeprazole; OME: omeprazole.
